# Age-related change in adult chimpanzee social network integration

**DOI:** 10.1093/emph/eoab040

**Published:** 2021-12-01

**Authors:** Nicole Thompson González, Zarin Machanda, Emily Otali, Martin N Muller, Drew K Enigk, Richard Wrangham, Melissa Emery Thompson

**Affiliations:** 1 Department of Anthropology, University of New Mexico, Albuquerque, NM 87131, USA; 2 Health Sciences Center, University of New Mexico, Academic Science Education and Research Training program, Albuquerque, NM 87131, USA; 3 Kibale Chimpanzee Project, Fort Portal, Uganda; 4 Department of Anthropology, Tufts University, Medford, MA 02155, USA; 5 Department of Human Evolutionary Biology, Harvard University, Cambridge, MA 02138, USA

**Keywords:** social isolation, comparative gerontology, social ties, age-related disease, senescence, embeddedness

## Abstract

**Background:**

Social isolation is a key risk factor for the onset and progression of age-related disease and mortality in humans. Nevertheless, older people commonly have narrowing social networks, with influences from both cultural factors and the constraints of senescence. We evaluate evolutionarily grounded models by studying social aging in wild chimpanzees, a system where such influences are more easily separated than in humans, and where individuals are long-lived and decline physically with age.

**Methodology:**

We applied social network analysis to examine age-related changes in social integration in a 7+ year mixed-longitudinal dataset on 38 wild adult chimpanzees (22 females, 16 males). Metrics of social integration included social attractivity and overt effort (directed degree and strength), social roles (betweenness and local transitivity) and embeddedness (eigenvector centrality) in grooming networks.

**Results:**

Both sexes reduced the strength of direct ties with age (males in-strength, females out-strength). However, males increased embeddedness with age, alongside cliquishness. These changes were independent of age-related changes in social and reproductive status. Both sexes maintained highly repeatable inter-individual differences in integration, particularly in mixed-sex networks.

**Conclusions and implications:**

As in humans, chimpanzees appear to experience senescence-related declines in social engagement. However, male social embeddedness and overall sex differences were patterned more similarly to humans in non-industrialized versus industrialized societies. Such comparisons suggest common evolutionary roots to ape social aging and that social isolation in older humans may hinge on novel cultural factors of many industrialized societies. Lastly, individual and sex differences are potentially important mediators of successful social aging in chimpanzees, as in humans.

**Lay summary:** Few biological models explain why humans so commonly have narrowing social networks with age, despite the risk factor of social isolation that small networks pose. We use wild chimpanzees as a comparative system to evaluate models grounded in an evolutionary perspective, using social network analysis to examine changes in integration with age. Like humans in industrialized populations, chimpanzees had lower direct engagement with social partners as they aged. However, sex differences in integration and older males’ central positions within the community network were more like patterns of sociality in several non-industrialized human populations. Our results suggest common evolutionary roots to human and chimpanzee social aging, and that the risk of social isolation with age in industrialized populations stems from novel cultural factors.

## INTRODUCTION

Social isolation leads to an increased risk of age-related morbidity, mortality and cognitive decline across a number of industrialized human populations [1, 2]. Equally, social ties curb the risk of mortality in a broad range of social animals [3, 4]. The social ties that individuals form with partners over time and the networks in which they are integrated are important sources of support, i.e. social capital, including access to tangible help, information and secure and stable environments [1, 4]. Despite the advantages of social integration, humans commonly shrink their network of social partners with age and reallocate social effort towards a small subset of partners [5–7]. A major goal in social gerontology has therefore been to understand the patterns that distinguish ‘successful’ from pathological social aging [5, 8].

Hypotheses for age-related declines in sociality in humans have focused on human-specific causes, such as shifts in cognitive-affective priorities with age that are driven by a perception of remaining lifetime [9], broken-down systems of extended family support in industrialized society [5], and/or significant life events that change social circles (e.g. retirement [7]). Humans, however, are not the only animals that exhibit decreased social integration with age (e.g. macaques, capuchins, lemurs, reviewed in ref. [10], yellow-bellied marmots [11]). Thus, holistic interpretations of social aging require a more generalizable framework, such as that offered by life history theory. Under such theory, individuals are predicted to use social behavior to adjust to physiological priorities and environmental challenges that vary by life stage and individual history. Key to this perspective is that social partners are a potential source of both stress and support [1, 4]. Because of tradeoffs in the costs and benefits of sociality, older individuals’ sociality may be driven by shifting reproductive priorities and/or energetically constrained by physiological senescence. Comparative studies are essential for evaluating this perspective because they help situate human behavior and biology in its evolutionary context. Chimpanzees, one of our closest evolutionary relatives, are a useful comparative model of social aging as they are long-lived and socially complex but occupy more tractable social networks and relatively few lifestyle and cultural confounds.

Recent evidence shows that male chimpanzees exhibit striking similarities to humans in how their dyadic friendships change with age [10, 12], suggesting shared evolutionary influences. Our present study examines patterns of social aging using a mixed-longitudinal behavioral dataset from wild chimpanzees. Our work builds on prior work by Rosati *et al*. [12] in two ways: (i) we incorporate network-wide measures of social integration, which may reveal different trends than direct social ties and (ii) we evaluate social network integration in both males and females, allowing us to determine whether social aging patterns occur consistently when the sexes occupy different baseline social profiles. We center our analysis on how suites of network measures can reveal evolutionarily relevant drivers of social aging (Tables 1 and 2 and Supplementary Material).

**Table 1.** Guide to individual network measures, where individual of interest is ‘ego’

Network measure	Functional term	Technical description
In	Social attractivity	Attention received
Degree		Number of partners that groom ego
Strength		Summed dyadic rates of ego’s grooming received
Out	Overt social effort	Attention given
Degree		Number of partners that ego grooms
Strength		Summed dyadic rates of ego’s grooming given
Betweenness^a^	Social role: bridging	Number of shortest paths between any two network members that pass through ego
Local transitivity	Social role: clique member	Proportion of ego’s partner that are also partners with each other
Eigenvector centrality	Embeddedness: influence and access to information	Individuals with high eigenvector centrality have many partners who themselves also have many partners

a All SNA measures from betweenness down are calculated with weighted and undirected edges.

**Table 2.** Guide to explanatory models of social aging tested in this study and their predicted changes in social integration

Model of social aging	Predictions
Sociosexual status	Dominance rank or sexual status drives variation in integration, where age did in models with age alone as a predictor.
Senescence constraints	All network measures of integration ↓ with age.
Added value	↑ Attention received and indirect connections (betweenness, embeddedness) with age.
Individual differences	Repeatable inter-individual differences explain significant amount of variation in integration, with or without age-effects.

### Social network data

Chimpanzees are a tractable comparative model for human social aging, in part, because they overcome common biases in human behavioral data (e.g. recall and social desirability biases, interactions limited to phone records [13]). For example, data from habituated non-human primates consist of extensive and direct observations of social behavior that are suitable for constructing accurate structural measures of social integration, which can be more powerful in predicting morbidity and mortality in humans relative to perceived experience [2]. As a further advantage, chimpanzee communities present clearly bounded social networks, where factors such as social and reproductive status, which are known to influence sociality, can be controlled for more easily than in more culturally complex human systems. In this study, we employ social network analysis (SNA) as a powerful and standardized tool to quantify structural features of individual social integration, with the advantage of incorporating indirect ties that situate individuals within groups as a whole (Tables 1 and 2 and Supplementary Material).

### Study system

We used SNA to measure age-related changes in social integration in wild, adult chimpanzees (*Pan troglodytes*) in the Kanyawara community in the Kibale National Park, Uganda. Chimpanzees live in large communities that are closed and they associate in a fission-fusion pattern which allows for inter-individual variation in social integration. Although chimpanzee social life lacks important components of human social networks such as marriage, nuclear families and an extended post-reproductive stage of life [14], chimpanzees do maintain strong ties with kin [15, 16]. They also have long lifespans (>60 years in the wild [17]) and experience age-related declines in physical condition [18]. Chimpanzees demonstrate stark differences in social tendencies between sexes. Males interact more frequently than females and remain in their natal communities for life, where they benefit from cooperative coalitions with other males to rise in dominance rank and access mates [19]. Females, in contrast, are less gregarious and less socially interactive than males [20], although this can vary somewhat with local ecology and community demographics [21]. Although female chimpanzees are less likely to form strong ties with one another than are males, strong female–female ties do occur [15]. Both males and females form linear dominance hierarchies based on competitive interactions, where high rank is associated with priority of access to fertile females for males [17], high-quality feeding areas and access to food for females [22, 23], and higher reproductive success in both sexes [22, 24]. As such, dominance rank represents a close approximation of socioeconomic status in humans in terms of social profiles, health and fitness disparities [3].

We evaluated age-related changes in males and females’ integration within grooming networks, quantified by seven social network measures (Tables 1 and 2 and Supplementary Material). Direct measures of social attractivity include in-degree and in-strength, quantifying the number of grooming partners and overall amount of grooming received, respectively. Out-degree and out-strength similarly characterize social effort as the number of grooming partners and total time spent grooming others. Other measures are ‘indirect’ quantifying an individual’s integration within the broader network. Social roles within the broader network include whether individuals interact within ‘cliques,’ i.e. among partners also connected with one another (local transitivity) and how often individuals bridge otherwise unconnected network members (betweenness). Lastly, we quantify how well-embedded individuals are in their network (eigenvector centrality). For a full explanation of the choice of network measures, including their functions and known changes with age in humans and other primates, see Supplementary Material.

Although multiple dimensions of social network integration allow for many combinations of results, we examined changes in social network integration for consistency with four explanatory models of social aging (Tables 1 and 2). First, because dominance rank and reproductive status vary with age and are both strong drivers of sociality [14, 25, 26], these factors may mediate apparent age-related changes in social integration. Under this sociosexual status model, we predict that age-related changes in sociality over the life course are specifically linked to changes in dominance rank and/or sexual status, but that age *per se* does not independently influence integration. Second and alternatively, senescence may pose physiological, physical or cognitive constraints on integration, which would lead to progressive social isolation and decreases in all integration measures. Third, age may confer added value to individuals in terms of either their attractivity as a social partner or their ability to make effective use of social relationships (akin to ‘prestige’ and social selectivity in humans). In this case, at least some aspects of integration will increase with age, such as greater attention received or indirect connections. Finally, because personality influences morbidity and mortality [1, 27], we examined the potential for individual differences to shape levels of integration over the life course, alone or in combination with age effects.

## METHODS

### Data collection

**Figure 1.**
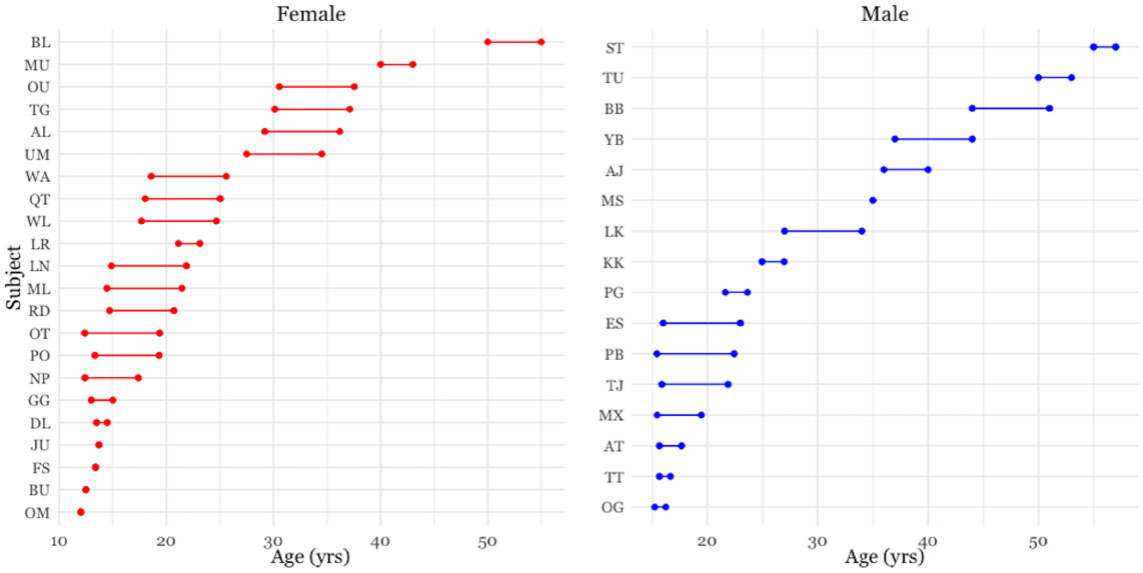
Age ranges of observation for each study subject (22 females and 16 males; 122 female-years, 78 male-years). Focal observations were continuous over each age window.

Data were collected on 38 permanent residents (22 females, 16 males) of the Kanyawara Community in the Kibale National Forest, Uganda from August 2009 to December 2017 (full Data collection methods and Ethical statement in Supplementary Material). Subjects included all individuals aged 12–57 years old (Fig. 1), beginning at the age when chimpanzees are socially independent from their mothers. Observers collected behavioral data during all-day focal follows, recording the subject’s activity and social partner(s) every 1 min and a scan of party membership every 15 min. Annual dyadic grooming rates were calculated as minutes of grooming standardized by minutes of shared party membership. In total, data consisted of 3371 focal follows, with subjects observed as focals for 133 ± 73 h per year (mean ± SD) and as party members during focals for 1033 ± 588 h per year.

### Analysis

**Figure 2.**
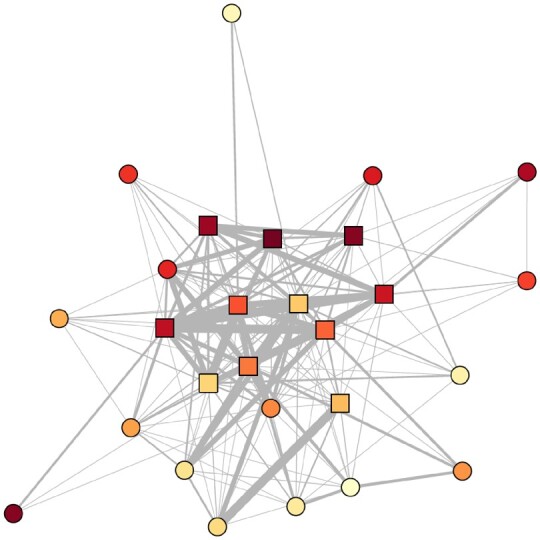
Sociogram of an annual network (year 2012). Males represented by square nodes and females by circles. Color of node darkens by individual age. Edges between nodes represent undirected grooming interactions, weighted by rates of dyadic grooming per time observed. Node layout determined by the Fruchterman-Reingold algorithm, where nodes with more and stronger direct edges appear nearer to one another. Individuals with fewer or weaker ties are thus placed at the periphery.

We used the R package igraph v. 1.2.6 [28] to create network graphs and measure the individual-level network integration (Fig. 2). Because inter- and intrasexual selective pressures have differentially shaped the form and function of male–male, female–female and male–female social relationships in chimpanzees, we evaluated integration within networks with sex compositions that captured these functionally distinct social realms. Namely, we calculated integration within grooming networks composed of both males and females (mixed-sex) or of all males or all females (same-sex; Supplementary Material). We calculated **in-degree, in-strength, out-degree** and **out-strength** for directed grooming networks and **local transitivity**, **betweenness** and **eigenvector centrality** in undirected grooming networks. All measures apart from in-degree and out-degree were weighted in an effort to capture variation in both numbers of social partners and frequencies of social interaction.

To evaluate changes in the network integration with age, we constructed general additive mixed models (GAMMs) in the R package mgcv v. 1.8-31 [29]. General additive models were useful for our age analysis because we expected social integration to vary over the life course in a non-linear fashion, as reproductive priorities and physiological constraints demonstrate non-monotonic changes with age [17, 18]. The curviness of non-linear relationships in GAMMs (smooths) is determined by the number of basic functions for each fixed effect, optimized for each model and effect (with mgcv::gam.check). All smooth parameters were estimated with restricted maximum likelihood. Each network integration measure was modeled as a response with either a Gaussian or Gamma error distribution and a log-link function, based on model diagnostics with the mgcv::gam.check function. We ran our models in three sets (Table 3). Sets 1 and 2 isolated the independent effect of age by controlling for dominance rank (set 1, mixed- and same-sex networks, Supplementary Tables S3 and S4) and the proportion of days when females exhibited maximal sexual swellings (set 2, mixed-sex networks, Supplementary Table S5; see Supplementary Material for calculations). In time swollen models, we included an interaction between female age and time swollen, as we expected females in estrus to be more attractive to males when they were older [26]. Set 3 examined the association with age only and was conducted to understand whether chimpanzees experience age-related changes in social integration, regardless of their cause (Supplementary Tables S7–S9). We compared the general effects of age in model set 3 with results of model sets 1 and 2, to evaluate whether dominance rank or reproductive status mediated age effects on integration (full method in Supplementary Material).

**Table 3.** GAMM compositions: testing effects of age on social integration independent of annual dominance rank and time swollen^a^

Approach	Network composition	Responses	Linear predictors and smooth terms
Rank-independent age effects	Mixed sex	In-degree, out-degree,^b^ in-strength, out-strength, local transitivity, betweenness, eigenvector centrality	Sex + s(age, by = sex, k) + s(rank, by = sex, k)
	Same sex	‘’	s(age, k) + s(rank, k)
Time swollen-independent age effects (females only)	Mixed sex	‘’	s(age, k) + s(rank, k) + s(time swollen, k) + ti(age, time swollen, k)
General age effects	Mixed sex	‘’	Sex + s(age, by = sex, k)
	Same sex	‘’	s(Age, k)

a All models included individual ID as a random effect: s(ID, bs = ‘re’).

b In-degree and out-degree calculated based on directed grooming networks, other measures on undirected networks.

Generalized additive models as implemented by the mgcv package are robust to concurvity [29], an issue similar to collinearity but for non-linear models. Thus, although male and female dominance rank, and female annual time swollen, were strongly related to age (Supplementary Table S1), estimates of their independent effects on integration were stable. Permutation methods were used for significance testing of the influence of predictors on integration measures (Supplementary Material). This method, where effect sizes are compared to those from models run on node-randomized permutations of observed data, reduces the risk of type I error that typically grows with multiple testing, and so avoids the need for correction of multiple comparisons [30]. Consistent inter-individual differences in social integration (repeatability) were evaluated by variance decomposition of each GAMM’s random effect of individual ID, identical to methods employed in linear models [31] and their significance calculated via permutation methods used in models of social aging (Supplementary Material).

## RESULTS

### Males

As expected, many aspects of social integration were predicted by male status. Higher ranking males were groomed by more male partners (Table 4, Supplementary Fig. S1), an effect that mediated an association between age and in-degree. Rank also influenced attractivity (in-degree), social effort (out-degree), and betweenness in mixed-sex networks, but this did not result in age-related changes in these measures (Supplementary Tables S3 and S8).

**Table 4.** Summary of results

Integration measure	Males (mixed sex)		Males (same sex)		Females (mixed sex)		Females (same sex)	
	Δ with age	IDE_obs_	Δ with age	IDE_obs_	Δ with age	IDE_obs_	Δ with age	IDE_obs_
In-degree	**⋅**	**⋅**	**⋅** ^a^	**⋅**	**⋅** ^b^	0.21 [98]	**⋅**	0.36 [99]
Out-degree	**⋅**	0.18 [96]	**⋅**	0.22 [100]	**⋅** ^b^	0.52 [100]	**⋅**	0.55 [100]
In-strength	**⋅**	0.37 [100]	**∩ [95]**	0.26 [96]	**⋅**	0.18 [100]	**⋅**	**⋅**
Out-strength	**⋅**	**⋅**	**⋅**	**⋅**	**↓ [100]**	0.21 [100]	**⋅**	0.13 [100]
Local transitivity	**↑[100]**	**⋅**	**⋅**	**⋅**	**⋅**	**⋅**	**⋅**	**⋅**
Betweenness	**⋅**	**⋅**	**⋅**	**⋅**	**⋅**	**⋅**	**⋅**	0.25 [95]
Eigenvector centrality	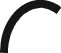 **[96]**	**⋅**	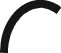 **[99]**	**⋅**	**⋅**	0.63 [100]	**⋅**	**⋅**

Age-related changes in social network integration, independent of dominance rank and time swollen (females). Icons describe significant relationships between age and a given network measure in GAMMs (see legend; full model results in Supplementary Tables S3–S4, S8–S9). Dots indicate a non-significant relationship with age. Significant repeatability of an integration measure is given as IDE_obs_ (observed deviance explained by individual ID in GAMM, full results Supplementary Table S6). Significance of the observed F statistic of age-related change and IDE_obs_ in GAMMs were evaluated by the % of 1000 statistics extracted from models on node randomized data that the observed statistics were greater than, noted in square brackets.

Integration measure ↑ = increases with age, ↓ = decreases with age, 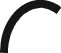 = increases and plateaus with age, **∩ =** increases in early to mid-adulthood and decreases in later adulthood.

a Rank mediates age effect on integration (Supplementary Tables S4 and S9, Fig. S1).

b Time swollen mediates age effect on integration (Supplementary Table S5 and S8, Fig. S2).

**Figure 3.**
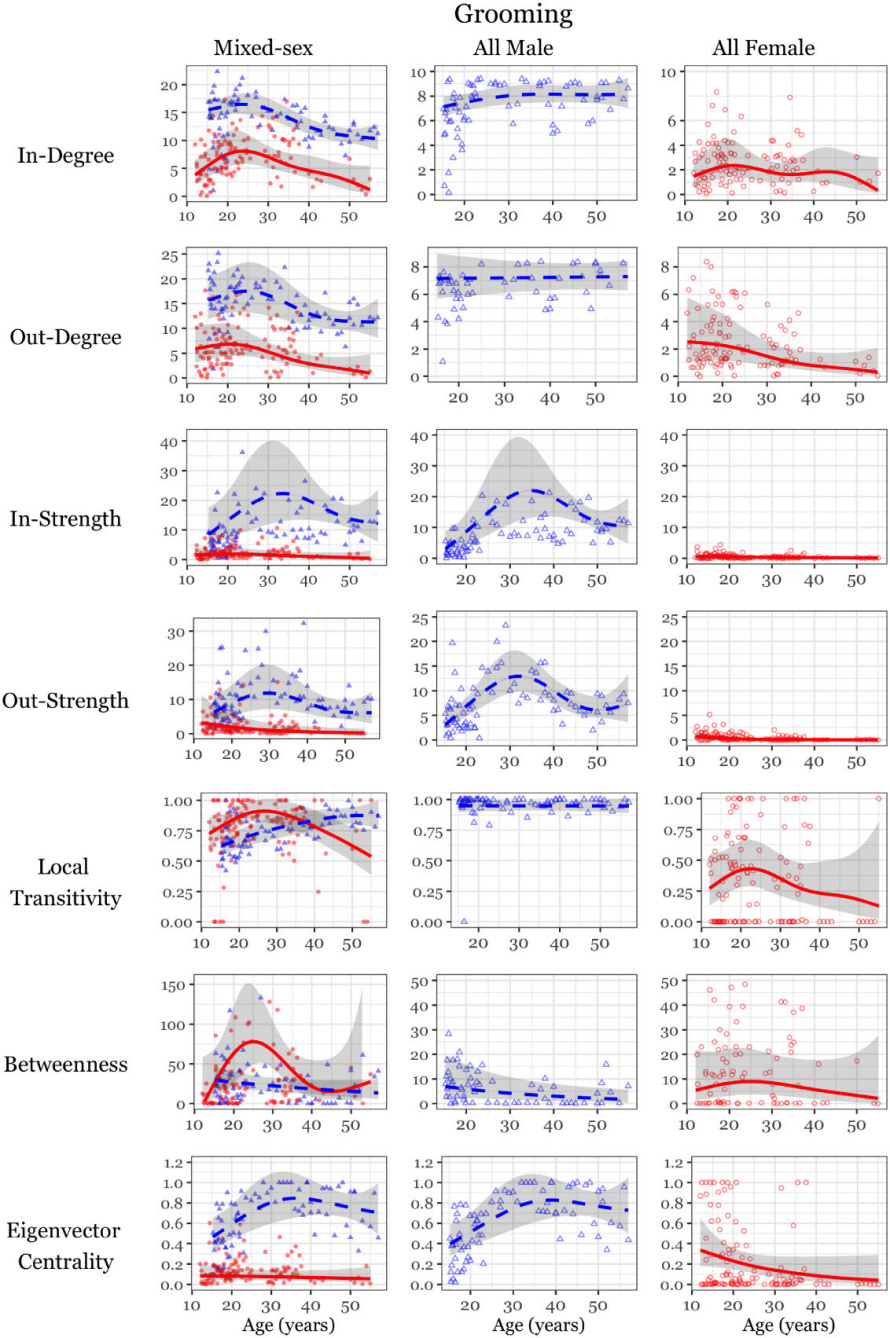
Social integration measures by age in mixed and same-sex grooming networks. Male data represented by blue triangles and blue dashed GAM smooth, female data represented by red circles and red solid GAM smooth. Smooths are conditional effects of age on social integration, controlling for rank, created using the R functions visreg and mgcv::gam within ggplot2.

Males also exhibited age-related changes in social integration that were independent of social status (Fig. 3, Table 4). First, older males received less grooming in all-male networks, after a peak in mid-adulthood (in-strength, Fig. 3, Table 4). Second, aging was associated with a linear increase in local transitivity for males in mixed-sex networks, meaning that each male’s grooming partners also frequently groomed one another (Fig. 3, Table 4). As transitivity did not change with age in the male-only network, this suggests that their increased ‘cliquishness’ in mixed-sex networks resulted from older males grooming with fewer females. Third, males’ embeddedness among partners (eigenvector centrality) changed with age in both mixed and same-sex networks (Fig. 3, Table 4). This relationship was such that the oldest males declined somewhat from a midlife peak in embeddedness but remained more central than younger males. Males also maintained highly repeatable inter-individual differences in their social effort (out-degree) and attractivity (in-strength, Table 4).

### Females

Relative to males, females displayed low levels of integration overall (Fig. 3, Supplementary Table S3). In years when females had a higher frequency of sexual swellings, in-degree and out-degree increased, albeit weakly, mediating apparent age-related declines in these measures (Table 4, Supplementary Fig. S2). That is, females appeared to have fewer grooming partners with age (in/out-degree, Fig. 3) because older females spent less time swollen (Supplementary Table S2). Dominance rank exerted little influence on female social integration, corresponding only with increased time being groomed (in-strength, Supplementary Fig. S1 and Table S5), and thus did not drive any age-related changes.

Independent of sociosexual status, age influenced females’ social effort (out-strength) in mixed-sex networks. Older females spent less time grooming in mixed-sex networks, but not in female-only networks (out-strength, Fig. 3, Table 4). Other network measures, including indirect measures, were not affected by female age. On the other hand, females showed repeatable inter-individual differences in most measures of network integration (Table 4).

## DISCUSSION

We used a mixed-longitudinal dataset to investigate whether aging influences social network integration in wild chimpanzees. Social aging patterns have been documented in humans and a handful of other primates [10], however, a unique goal of this study was to discriminate how aging influences social integration independently of its effects on social and reproductive status. Our results were consistent with prior evidence indicating that dominance rank and female reproductive status influence social relationships in chimpanzees [15, 16, 32]. Nevertheless, we identified some specific effects of aging itself on social integration (Table 5). Older males were more embedded in networks (high centrality) and more cliquish than younger males (high local transitivity), whereas females, who were consistently less integrated than males, declined in social effort with age (low out-strength). Our findings suggest that physical and/or cognitive aging processes do not strongly constrain integration in chimpanzees. Although senescence may exacerbate the social constraints already experienced by chimpanzee females, aging itself does not appear to promote their social isolation. Additionally, males and females both demonstrated individually stable social phenotypes, suggesting that like humans, individual chimpanzees may be predisposed to more or less successful aging trajectories [8]. Here, we discuss patterns of male and female social aging separately regarding our four explanatory models, compare patterns to other non-human primates and consider their implications for human social aging and age-related disease.

**Table 5.** Summary of evidence consistent and inconsistent with 3 models of social aging

Model of social aging	Male	Female
Sociosexual status	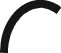 **In-Degree** ^SS^ **with dominance rank mediates age effect.**	**↑ In-Degree** ^MS^ **& ↑ Out-Degree** ^MS^ **with time swollen mediates age effect.**
Senescence constraints	**∩ In-Strength with age** ^MS^	**↓ Out-Strength^MS^**
	↑ Transitivity^MS^ and sustained Embeddedness into old age.	
	No age-related changes in social effort.	
Added value	**High embeddedness sustained in old age.**	
	∩ In-Strength with age^MS^	
Individual differences	**Social attractivity and social effort measures are repeatable.**	**Majority of network measures are highly repeatable.**

Evidence consistent with model is in bold, inconsistent is unbolded.

MS Change occurs in mixed-sex networks only.

SS Change occurs in same-sex networks only.

### Role of sociosexual status in social aging

Dominance rank often shifts with age, because of changes in physical power and seniority [25]. Male chimpanzees experience their peak social rank in early to mid-adulthood, declining thereafter. By contrast, female chimpanzees experience a reliable increase in social rank with age [33]. In addition, female associations with males are strongly affected by sexual condition [26, 32], thus age-related changes in fecundity and attractiveness are likely to influence patterns of female social aging. As expected, sociosexual factors did have an impact on social network measures in our study; however, there were few cases where age-related changes in status were sufficient to yield an age effect. Dominance rank explained age-related changes in the number of male partners from whom males received grooming (in-degree), whereas sexual status accounted for the changes in females’ grooming partners in mixed sex networks (in-/out-degree).

### Social aging in males

As they aged, males elicited less grooming from other males (low in-strength) than predicted by declining rank alone. Despite this, males were able to maintain high embeddedness within both mixed-sex and all-male networks. In other words, without increasing the number or strength of social ties, older males were more socially central than younger adults. As older chimpanzees of both sexes are found less often in association with others [12, 34], males’ ability to maintain central network positions is even more striking and suggests selective maintenance of valuable ties, i.e. relationships with well-connected partners. Although our evidence cannot distinguish between possible mechanisms, such maintenance could be accomplished by older males reallocating social effort towards highly connected individuals and/or by their increased attractivity as social partners to such individuals.

In a recent publication on dyadic social relationships among Kanyawara males, social selectivity increased with age [12]. Although aging did not affect the number of bonds that males formed, older males had more ‘mutual’ friendships characterized by equitable investment of both partners. This focus on valuable relationships perhaps contributes to the pattern of increased embeddedness observed here. The use of social ‘ties’ (i.e. any affiliative relationship) in social network analyses differs from evaluation of social ‘bonds’ (i.e. particularly strong relationships), so analyses of ties are less sensitive to the skew in allocation of social effort to particular partners. Indeed, an interesting characteristic of the male chimpanzee social network is how well connected the entire cohort of adult males is, such that selectivity must occur via redistribution of social behavior rather than winnowing of social partners. Social selectivity could also lead to the increased transitivity observed among older males. However, this pattern was only detected in mixed-sex networks, suggesting that it instead reflects reductions in relationships with females.

A second possible pathway for older males’ high embeddedness is an increase in social attractivity. Because we found that older males received less grooming (low in-strength) than did middle-aged males, this mechanism of sustained integration is not strongly suggested. Nevertheless, older males maintained their number of incoming ties (in-degree), leaving the possibility that males sustained embeddedness by being groomed disproportionately from particularly well-connected individuals. In some long-lived species, including elephants and orcas, older individuals are central to communities because they have accumulated valuable socioecological knowledge [35–37]. Future studies may examine fine-scale shifts in chimpanzee male attractivity based on the connectedness and dominance rank of social partners.

Greater embeddedness, as measured by network centrality, is presumed to benefit individuals with social capital that extends beyond that of direct ties [38]. One basic form of capital in indirect ties could be transitively conferred tolerance between individuals A and C in the presence of connecting individual B. In humans, the benefits of indirect ties are often framed in terms of access to information or resources [38]. While communication of information is likely more limited in primates, chimpanzees do exhibit cultural transmission of behavior that appears to propagate through social networks (e.g. [39]). Chimpanzee males are the primary participants of large-scale cooperative behaviors such as hunting and territorial patrols. It is possible that cohesion among indirect ties explains mutual participation when not all individuals are directly and strongly connected, similar to a domino effect. For example, among chimpanzees in the Taï Forest, Côte d’Ivoire, individuals were more likely to participate in an intergroup encounter when a single bond partner was already involved [40].

### Social aging in females

Females were less integrated than males by most measures, corroborating prior work that female chimpanzees in most populations are less gregarious and have fewer social bonds than males [21]. Females’ sole change in sociality with age *per se* was a decrease in their social effort (out-strength). As general constraints, female chimpanzees experience particularly strong feeding competition when in social groups [20] and association with males both exposes females to aggression via sexual coercion [34, 41] and reduces female foraging efficiency [42]. Although males and females decline in physical condition at the same rate [18], aging may nevertheless exacerbate these constraints on female sociality. For example, females become more sexually attractive to males with age [26], as evidenced in this study by older females’ increased grooming received when sexually swollen (in-strength, Supplementary Fig. S3). Further, females’ number of companion offspring increase with age, leading to greater vulnerability to competition in large foraging parties [34]. Consistent with this reasoning, older females reduced social effort within mixed but not same-sex networks, indicating reduced interactions with males. This change in effort did not, however, significantly influence females’ already low social embeddedness.

### Significance of individual effects on integration

Kanyawara chimpanzees maintained stable between-individual differences in several dimensions of social integration (Table 4), e.g. certain chimpanzees consistently gave more grooming than others, similar to chimpanzees in the Taï Forest [43]. Thus, if social integration is important to health in chimpanzees, as it is in humans and many other species, individuals’ social phenotypes could be more or less conducive to successful aging [8]. As individual differences explained more variation in female social integration than did sociosexual status or age, further examination of the attributes driving female chimpanzees’ differences in social integration is well warranted.

### Comparison with non-human primates

Social aging is a common phenomenon in wild primates and is usually associated with a reduction in social integration with age, though these patterns vary in species- and sex- specific ways [10]. Many such studies are on females of female-bonded species, such as macaques and baboons, where decreases in female social integration with age may be detected in part because females are so highly integrated in young adulthood. In chimpanzees, males are the more socially integrated sex and, here, did not suffer reduced integration with age. Three factors may account for this difference. First, in female-bonded species, ties are formed preferentially with kin, therefore social network positions are likely biased by kin availability and compromised by the deaths of aging kin [44]. Male chimpanzees are only marginally biased toward kin (e.g. [45]) and maintain a wide array of grooming ties, readily replacing them over time [46]. Second, chimpanzees have extended lifespans relative to cercopithecines, therefore males perhaps employ strategies to maintain social ties in their prolonged old age that are less advantageous in other primate species. Notably, in the Kanyawara community, male chimpanzees continue to sire offspring well past their physical prime, and as in humans, may use coalitionary support to do so [17]. Lastly, contrasts between our results and those of other primate studies could stem from differences in analytical approach. As recommended by Farine and Whitehead [30], we used permutation tests to determine the significance of patterns, whereas many other studies do not. This approach rigorously controls for the dyadic non-independence of network measures as response variables and likely produces more conservative estimates of social change with age.

### Comparisons to and implications for human social aging

Key patterns of social aging in chimpanzees were consistent with those in industrialized human populations. Like industrialized humans, male and female chimpanzees decreased their direct social engagement with age, with their highest levels of interaction in early adulthood [6, 7, 47]. Further, male chimpanzees participated in tighter social cliques, rather than increasingly bridging otherwise unconnected partners, like many men [5, 9]. However, unlike most men in industrialized societies, chimpanzee males sustained high levels of embeddedness into old age. Further, chimpanzees’ sex differences in social aging were largely opposite to that observed in industrialized populations, where women consistently have larger networks than men after early adulthood [5, 48].

Where Kanyawara chimpanzees contrasted with industrialized humans, they aged more similarly to humans in non-industrialized settings, where social networks are primarily based within small communities. Although data on social aging from non-industrialized societies are sparse and preclude robust comparisons, several similarities are apparent. Men in non-industrialized societies, such as in Tsimane forager-horticulturalists and Nyangatom agro-pastoralists, often retain significant social capital in old age, similar to male chimpanzees [49]. Further, female chimpanzees’ low social integration relative to males resembles the situation of women in some patrilocal and non-industrialized societies that disperse at marriage and are limited in replacing kin relationships with new non-kin partners [50, 51]. For example, in Himba semi-nomadic pastoralists, women are often hindered in their travel to visit kin for social support because of mate-guarding within their marriage [51]. Among the Tsimane and nomadic Saami, women also face trade-offs between having large, cooperative social networks and attending to duties of intra-household labor and childcare [52, 53]. In each case, women are socially limited by male reproductive tactics and their reproductive priorities. Future studies on age-related changes in sociality in diverse populations of humans and chimpanzees will allow even greater inferences into how ecological variability in gender roles shapes social aging and into the nature of humans’ ancestral social environments.

While humans and chimpanzees live in quite different social contexts, similarities in social aging patterns (see also [10, 12]) suggest that human social aging may be influenced by evolutionary forces that pre-date our particular cognitive capacities and social environments. Thus, there is a need to extend social aging theory to consider patterns shared with other species, such as age-related shifts in the costs and benefits of social interactions [12].

### Implications for human age-related disease

Although social integration is well linked to fitness in non-human primates [3, 4], whether social integration moderates age-related declines in physical health in non-human primates is currently an open question. Although we did not yet test these effects here, our evidence supports the view that age-related reductions in social engagement need not lead to pathological social isolation. Indeed, we hypothesize that these changes reflect broader life history strategies to accommodate shifting costs and benefits of social behavior with aging. Following parallel logic to evolutionary mismatch theory, as it has been applied to physical health, we suspect that social environments of the past that sustained embeddedness and social status are now less common in industrialized human society, making social isolation in old age more prevalent. Industrialized societies typically differ from non-industrialized societies in important ways: a lack of deference to older people [54], communities that are less cohesive across the lifespan [55], and gender norms that promote male stoicism and independence, as opposed to tolerance and cooperation [56]. A relatively stable community alone could preserve chimpanzees’ network size and allow male social knowledge and female social status to accrue. Insights gained from further comparative research across human populations and with closely related species can inspire and support the rationales of certain social interventions for older people, such as prioritizing stability and control in older adults’ social environments over a manufactured sense of belonging or introduction of new social ties [1, 47].

## Supplementary data

Supplementary data is available at *EMPH* online.

**Conflict of interest:** None declared.

## Data availability

Relevant data and scripts for analysis are publicly available in author NTG’s GitHub page at https://github.com/Gavago/Social-aging-in-wild-adult-chimpanzees.git.

## Supplementary Material

eoab040_Supplementary_Data
