# Supplementary material for: Age-related change in adult chimpanzee social network integration
Source: Evol Med Public Health. 2021 Dec 1;9(1):448–59. doi: 10.1093/emph/eoab040 (PMC8697844; doi:10.1093/emph/eoab040)
Supplement: eoab040_Supplementary_Data [file eoab040_supplementary_data.zip › 2._supplemental_text_and_tables_social_aging_11.6.2021 ...docx]

**Supplemental Background**

***Justification of Social Network Measures: functions and changes with age***

Social network analysis has the distinct advantage of providing individual measures of integration based on either direct or indirect ties, with the latter situating individuals within groups as a whole (Table 1). The overall number of direct social partners an individual has (i.e. **degree** centrality**)** represents its range or flexibility in possible sources of social support and resources (Donald & Ware, 1984; Thompson, 2019). Greater frequency of contact or associatio n with partners (i.e. **strength** or intensity of social ties), indicates individual gregariousness and the presence of preferential relationships that can predict reliable support (Bray & Gilby, 2020; Granovetter, 1983; James, 2000; Mitani, 2009; Young et al., 2014). In humans, although degree generally decreases with age (Cornwell et al., 2008; David-Barrett et al., 2016; English & Carstensen, 2014; Fung et al., 2001; Wrzus et al., 2013), strength does not always follow the same pattern, sometimes decreasing and sometimes remaining the same, indicating a relative increase among a smaller set of social partners (Carstensen, 1992; Cornwell et al., 2008). Directional measures of degree/strength further tease apart overt forms of individual social attractivity vs. social effort, or attention received vs. given. In Barbary macaques, for example, adult females maintain the same number of groomers and amount of grooming received as they age (**in-degree** and **in-strength**), but reduce their overt social effort by grooming fewer individuals less often (out-degree and out-strength, Almeling et al., 2016). Across animals, both social attractivity and effort change with age. For example, older individuals sometimes attract more attention because of their experience, including greater political knowledge (men, Glowacki & von Rueden, 2015; von Rueden et al., 2008), ecological knowledge (female orcas, elephants, and bonobos, Brent et al., 2015; McComb et al., 2001, 2011; Tokuyama & Furuichi, 2017), or reproductive parity (female chimpanzees, Anderson, 1986; Muller et al., 2006). Social effort, on the other hand, often decreases with age in many primates (reviewed in Machanda & Rosati, 2020), possibly because older and senescing individuals are simply less able to physically compete, a direct cost of sociality (Emery Thompson et al., 2020; Silk, 2007).

In humans, social roles are positions held within a group that involve both direct and indirect group ties. Roles in humans are thought to promote health by increasing one’s sense of identity and purpose (Cornwell et al., 2008; Holt-Lunstad et al., 2010) and potentially mirror several aspects of animal social behavior that similarly promote homeostasis and environmental stability (Matthews & Tye, 2019). In SNA, one measure of social role is participation in cliques, i.e. when one’s contacts interact with one another (**local transitivity**, Table 1). When social contacts form cliques it increases the likelihood that cooperation and reciprocity will ensue (Sosa et al., 2020), creating secure environments where information can be triangulated and where resources such as food and vigilance can be pooled (Cornwell et al., 2008; Hanneman & Riddle, 2005). A second measure of social role, and one often inversely related to transitivity, is an individual’s ability to bridge disparate cliques or otherwise unconnected individuals (**betweenness centrality**; Cornwell et al., 2009; Hanneman & Riddle, 2005). The benefit of bridging otherwise unconnected individuals is to uniquely access and broker information and/or to have access to distinct pools of resources (Brent, 2015; Keating et al., 2005). In dolphins (*Tursiops spp.*), for example, highly ‘between’ individuals possess greater ecological knowledge and are key in facilitating cohesion (Lusseau & Newman, 2004), and decision-making in communities (Lusseau, 2007). No human or non-human animal studies have yet examined age-related variation in social roles measured as local transitivity or betweenness *per se*. However, people’s increased participation in religious and volunteer organizations and focus on few, close social contacts in late adulthood suggests that humans do increase in local transitivity with age (Bhattacharya et al., 2016; Carstensen et al., 1999; Wrzus et al., 2013). Limited research indicates that humans have little to no tendencies to bridge different partners in old age (Cornwell et al., 2009; Wen Yuan et al., 2017).

Lastly, social “embeddedness” is a fundamental concept in the social determinants of health literature, highlighting that individuals derive social capital from their position within a global network of indirect ties, or “friends of friends”, including access to information and social norms (Carstensen et al., 1999; Coleman, 1988; Cornwell et al., 2008; Keating et al., 2005; Stowe & Cooney, 2015). Although widely referenced (e.g. Coleman, 1988; Granovetter, 1985), embeddedness *per se* is rarely quantified in human health studies, but can be well captured in SNA as **eigenvector centrality** (Andersen, 2013; hereafter, centrality, Table 1). High measures of centrality derive from an individual’s many and strong social ties and those of their direct contacts (Sosa et al., 2020). In non-human animals, centrality corresponds with greater food discovery (Paridae songbirds, Aplin et al., 2012), and has been shown to decrease with age in female yellow-bellied marmots (Wey & Blumstein, 2010), and in some primates (Barbary macaques, Rathke & Fischer, 2021) but not all of those examined (rhesus macaques, Liao et al., 2018). In some species, embeddedness corresponds with decreased parasites and infection (Balasubramaniam et al., 2016; Duboscq et al., 2016), however, under some circumstances it can lead to greater pathogen exposure (Nunn, 2012; Page et al., 2017). In humans, embeddedness is thought to decline with age alongside shrinking social networks (Cornwell et al., 2008).

**Supplemental Methods**

***Ethical statement***

The Institutional Animal Care and Use Committees of Harvard University and the University of New Mexico approved of this study’s data collection protocol. All research was conducted in compliance with Ugandan law, with research permissions granted by the Uganda Wildlife Authority, Uganda National Council for Science and Technology, and Makerere University Biological Field Station.

***Data collection***

The Kanyawara community of wild chimpanzees lives in the northern part of Kibale National Park, Uganda. From August 2009 to December 2017, pairs of field assistants of the Kibale Chimpanzee Project conducted focal follows of individual chimpanzees, wherein they attempted to follow the same chimpanzee (and that chimpanzee’s associates) through the entire active period from waking to nesting (mean ± sd = 9.8 ± 2.7 hrs per follow, N = 3371 follows). Focals were selected based on which individuals were located on a given day, prioritizing those who had been followed less recently or less frequently. If a focal was lost, another was chosen, if possible, to finish the observation day. One observer collected party composition data (all individuals within 50 m of any other) via instantaneous scan sampling every 15 minutes, while a second recorded the focal individual’s activity (e.g. resting, grooming, feeding) each minute

and the ID(s) of its social partner(s). The average chimpanzee was a focal subject for 133 ± 73 hours per year (130 ± 78 F, 138 ± 63 M) and a party member for 1033 ± 588 hours per year (937 ± 531 F, 1184 ± 642 M; annual values Table S1). Importantly, within subjects, no annual measure of social integration in any network was, on average, correlated with annual observation time as a focal or party member (subjects observed ≥ 3 years N = 30, range of average Spearman’s rho for within-individual correlations -0.28 – 0.57, all p > 0.28).

The study examined social integration in the 22 female and 16 male adults that permanently resided in the Kanyawara community between 2009 to 2017, for a total of 200 unique chimp-years. Networks were calculated on an annual basis, but because focal data collection started late in 2009, we combined data from 2009 and 2010. Social networks included only adult individuals, including males ≥ 15 years and females ≥ 12 years. Members ranged from 12 – 57 years old, with an average age of 26.5 +/- 11.6 years (mean +/- sd), and each member contributed to 1 – 8 years of networks, with an average 5.26 +/- 2.7 years (Fig. 1). Individuals were included as annual network members if present in the community for ≥ 6 months of the year (where absence was related to their pre-immigration status or death), and if observed either > 50 hours as a focal or > 100 hours as a party member during focals. These criteria led us to omit only 15 insufficient chimp-years, resulting in full adult networks that ranged from 22 to 27 individuals, male networks from 8 to 11 individuals, and female networks from 14 to 17 individuals.

***Calculation of covariates: grooming rates, annual age, dominance rank, and time swollen***

We calculated dyadic **grooming rates** by first summing the number of 1 min focal point samples throughout the calendar year when the dyad members were observed grooming. We then controlled for the dyad members' opportunity to interact by dividing grooming minutes by the sum of 15 min point samples that the dyad was seen in the same party while one was a focal subject, yielding grooming min/min in the same party.

We measured annual **age** at the mid-year (July 1) for all subjects. Birthdates of natal community members born after 1987 were known to within one year. Birthdates of individuals born before 1987 (most first encountered in 1983) were estimated based on body size, if immature, or by signs of relative aging, including body hair and presence of dependent offspring (see Muller & Wrangham, 2014). Immigrant, nulliparous females were assigned an age of 13, the average age when natal females are seen to disperse from the community. To calculate individual annual dominance **rank,** we averaged daily dominance ranks within sex-specific dominance hierarchies across one year. Daily dominance ranks were based on Elo ratings informed by decided agonistic interactions, as described in Emery Thompson *et al.* (2020), and standardized relative to number of individuals in the hierarchy (1 = highest rank, 0 = lowest rank). Lastly, to control for changes in reproductive activity with age, we calculated the proportion of observation days in a given year that a female was seen with a maximally tumescent swelling (**time swollen**). Mating primarily occurs when females are in this state (Muller & Wrangham, 2004), and associations with males consequently increase.

***Assessing significant changes in integration with age in GAMM models***

To control for dyadic non-independence in network data, we tested the significance of patterns of social integration related to age, sex, rank, and reproductive status in GAMM models by creating 1000 randomized versions of each network, where node attributes such as sex, age, rank, time swollen (among females alone), and ID were assigned randomly within years (Croft et al., 2011; Farine & Whitehead, 2015; Weiss et al., 2021). Node randomization preserved, and thus controlled for, annual variation in network size, sex and age composition, and potential stability in individual social tendencies. We ran our original models on these randomized data sets 1000 times each and extracted the estimated F statistics of the smooths of interest (e.g. age, rank, time swollen, age * time swollen) and linear coefficients of the categorical predictor “sex”. We then calculated the proportion of randomized F statistics and linear coefficients that fell below the observed models’ F statistic and coefficient, where proportions ≥ 0.95 indicated a significant pattern in the smooth term and ≥ 0.95 and ≤ 0.05 indicated a significantly positive or negative effect of the categorical predictor. Permutation methods, such as the one we employ, are strongly advocated to evaluate the significance of node level attributes on network measures because they control for the dyadic non-independence of network data (Croft et al., 2011; Farine & Whitehead, 2015; Weiss et al., 2021). Several other studies do not employ such permutation methods when examining age-related changes in animal social network integration (e.g. Goldenberg et al., 2014; Rathke & Fischer, 2021; Wey & Blumstein, 2010). Consequently, the results of our analyses are likely to be relatively conservative estimates of patterns of integration over time.

***Assessing age-effects mediated by sociosexual status in GAMM models***

To evaluate where dominance rank and/or reproductive status mediated age-effects on integration, we triangulated the results of our model sets relating age, sociosexual status, and social integration, similar to the “Difference Method” (VanderWeele, 2016). Specifically, when age was related to a measure of integration in age-alone models (Tables S8-9), we examined whether age maintained its relationship with integration when controlling for dominance rank (Tables S3-4) and both dominance rank and time swollen (females mixed-sex networks, Tables S5). We determined status to mediate the age effect on integration if 1) age was no longer associated with integration in models that controlled for sociosexual status and 2) either element of sociosexual status did itself associate with integration. This conclusion was supported by the strong relationships between age and dominance rank, and age and time swollen (Table S2).

***Calculating repeatable inter-individual differences***

To evaluate the individual differences model of social aging, we measured the consistency of individual differences (i.e. **repeatability**) in each social integration measure. We calculated a repeatability statistic by partitioning the deviance explained by individual intercept (ID) in each GAMM, following methods for generalized linear models (Nakagawa et al., 2017; Schielzeth & Nakagawa, 2020). In this approach, deviance explained is used as a coefficient of variation, similar to the R^2^ in linear models, that is generalized and appropriate for GAMs (Wood, 2017). We evaluated the significance of the repeatability statistic by comparing the observed deviance explained by individual ID to 1000 deviances explained by ID in models of node-randomized data, i.e. data with randomized attributes of rank, time swollen, and ID, within years, network behavior, network type, and individual sex. An integration measure was significantly repeatable if its repeatability statistic was ≥ 95% of its random statistics. Because of large sex differences in social tendencies, we modeled male and female repeatability separately, and controlled for annual rank, and annual time swollen (for females only in mixed sex networks) as fixed effects. Significantly repeatable inter-individual differences in integration in the absence of age effects in GAMMs would indicate variation in integration resulting primarily from individual traits, whereas repeatable differences in combination with an age effect on integration would represent differences in the extent of individual integration (intercept) within an overall age-related pattern.

**Table S1.** Average annual observation times per subject as focal or party member during focal follows.

| **Year** | **Sex** | **Mean ± sd focal hours per subject** | **Mean ± sd party hours per subject** |
| --- | --- | --- | --- |
| 2010* | F | 133.9 ± 118.9 | 922.6 ± 489.9 |
| 2010* | M | 153.2 ± 50.3 | 1248.3 ± 411.7 |
| 2011 | F | 66.3 ± 40.4 | 559.5 ± 169.7 |
| 2011 | M | 70 ± 17.7 | 752 ± 170.4 |
| 2012 | F | 85.5 ± 68.6 | 481.3 ± 181.4 |
| 2012 | M | 96.5 ± 37.1 | 504.1 ± 169.7 |
| 2013 | F | 116.5 ± 48.1 | 779.4 ± 219.4 |
| 2013 | M | 116.3 ± 44.2 | 911.4 ± 196.5 |
| 2014 | F | 137.4 ± 64.6 | 699.5 ± 216.3 |
| 2014 | M | 102.3 ± 45.1 | 743.8 ± 232.3 |
| 2015 | F | 160.9 ± 59.4 | 1808.3 ± 534.3 |
| 2015 | M | 180.5 ± 54.7 | 2289.7 ± 581.3 |
| 2016 | F | 186.8 ± 76.8 | 984.9 ± 429.3 |
| 2016 | M | 219.4 ± 48.6 | 1598.7 ± 424.7 |
| 2017 | F | 152.3 ± 64.8 | 1233.1 ± 391.1 |
| 2017 | M | 175.4 ± 37.3 | 1634.1 ± 455.4 |

*2010 = Aug-Dec 2009 & all 2010 combined

**Table S2.** Significant relationships in GAMM models between male and female age and annual dominance rank (Elo scores) and female age and annual time swollen (N females = 22 individuals, 122 female-years; N males = 16 individuals, 78 male-years). Significance evaluated with model P values. Male ranks showed a concurve pattern with age. Female ranks rose and plateaued with age. Female time swollen decreased linearly with age.

| **Response** | **Predictor** | **F** | **P value** |
| --- | --- | --- | --- |
| Annual dominance rank | female age | 29.6 | < 0.001 |
|  | male age | 60.2 | < 0.001 |
| Annual time swollen | female age | 19.4 | < 0.001 |

**Table S3.** GAMM models for all integration measures in mixed-sex grooming networks. Significant effects in bold with *. DE = total model deviance explained. Significance of the categorical variable sex and smooth terms age and rank evaluated by the proportion of ßs and F statistics drawn from randomized networks that the observed ß estimate and F statistic are greater than.

| **Response** | **Network sex** | | **DE** | **Predictors** | **F_obs_ of smooths** | | **ß_obs_ of sex(M)** | | **F_obs_ > F_ran_** | **ß_obs_ > ß_ran_** | |  |
| --- | --- | --- | --- | --- | --- | --- | --- | --- | --- | --- | --- | --- |
| In-Degree | mixed | | 0.75 | **sex** |  | | **0.61** | |  | **1*** | |  |
|  |  | |  | **female age** | **5.46** | |  | | **0.99*** |  | |  |
|  |  | |  | male age | 3.77 | |  | | 0.93 |  | |  |
|  |  | |  | **female rank** | **4.83** | |  | | **0.97*** |  | |  |
|  |  | |  | male rank | 3.28 | |  | | 0.94 |  | |  |
| Out-Degree | mixed | | 0.87 | **sex** |  | | **0.94** | |  | **1*** | |  |
|  |  | |  | **female age** | **4.87** | |  | | **0.98*** |  | |  |
|  |  | |  | male age | 3.01 | |  | | 0.91 |  | |  |
|  |  | |  | **female rank** | **6.68** | |  | | **0.99*** |  | |  |
|  |  | |  | **male rank** | **3.79** | |  | | **0.95*** |  | |  |
| In-Strength | mixed | | 0.78 | **sex** |  | | **1.28** | |  | **1*** | |  |
|  |  | |  | female age | 0.88 | |  | | 0.44 |  | |  |
|  |  | |  | male age | 5.59 | |  | | 0.9 |  | |  |
|  |  | |  | female rank | 0.22 | |  | | 0.22 |  | |  |
|  |  | |  | male rank | 5.21 | |  | | 0.93 |  | |  |
| Out-Strength | mixed | | 0.71 | **sex** |  | | **1.54** | |  | **1*** | |  |
|  |  | |  | female age | 3.52 | |  | | 0.82 |  | |  |
|  |  | |  | male age | 2.54 | |  | | 0.73 |  | |  |
|  |  | |  | female rank | 1.94 | |  | | 0.75 |  | |  |
|  |  | |  | **male rank** | **6.03** | |  | | **0.97*** |  | |  |
| Local Transitivity | mixed | 0.26 | | sex |  | | -0.07 |  | | | 0.02 | |
|  |  |  | | **female age** | **5.22** | |  | **0.99*** | | |  | |
|  |  |  | | **male age** | **17.31** | |  | **1*** | | |  | |
|  |  |  | | **female rank** | **3.39** | |  | **0.95*** | | |  | |
|  |  |  | | male rank | 1.23 | |  | 0.68 | | |  | |
| Betweenness | mixed | 0.26 | | sex |  | | 0.32 |  | | | 0.93 | |
|  |  |  | | female age | 2.86 | |  | 0.77 | | |  | |
|  |  |  | | male age | 0.91 | |  | 0.43 | | |  | |
|  |  |  | | female rank | 1.27 | |  | 0.54 | | |  | |
|  |  |  | | male rank | 0.21 | |  | 0.2 | | |  | |
| Eigenvector Centrality | mixed | 0.87 | | **sex** |  | **1.43** | |  | | | **1*** | |
|  |  |  | | female age | 2.06 |  | | 0.8 | | |  | |
|  |  |  | | **male age** | **4.92** |  | | **0.96*** | | |  | |
|  |  |  | | female rank | 2.46 |  | | 0.86 | | |  | |
|  |  |  | | male rank | 0.44 |  | | 0.39 | | |  | |

**Table S4.** GAMM models for all integration measures in same-sex grooming networks. Significant effects in bold with *. DE = total model deviance explained. Significance of the smooth terms age and rank evaluated by the proportion of F statistics drawn from randomized networks that the observed F statistic is greater than.

| **Response** | **Network sex** | | **DE** | **Predictors** | **F_obs_ of smooths** | **F_obs_ > F_ran_** |
| --- | --- | --- | --- | --- | --- | --- |
| In-Degree | same | | 0.42 | female age | 2.13 | 0.84 |
|  |  | |  | female rank | 1.35 | 0.68 |
| In-Degree | same | | 0.52 | male age | 2.18 | 0.88 |
|  |  | |  | **male rank** | **5.71** | **1*** |
| Out-Degree | same | | 0.42 | female age | 2.13 | 0.79 |
|  |  | |  | female rank | 1.35 | 0.65 |
| Out-Degree | same | | 0.52 | male age | 0.02 | 0.09 |
|  |  | |  | **male rank** | **3.34** | **0.95*** |
| In-Strength | same | | 0.28 | female age | 2.5 | 0.66 |
|  |  | |  | female rank | 0.36 | 0.22 |
| In-Strength | same | | 0.7 | **male age** | **5.77** | **0.95*** |
|  |  | |  | male rank | 0.02 | 0.07 |
| Out-Strength | same | | 0.28 | female age | 2.5 | 0.84 |
|  |  | |  | female rank | 0.36 | 0.4 |
| Out-Strength | same | | 0.35 | male age | 6.31 | 0.92 |
|  |  | |  | male rank | 0.27 | 0.34 |
| Local Transitivity | same | | 0.13 | female age | 1.64 | 0.77 |
|  |  | |  | female rank | 0.48 | 0.36 |
| Local Transitivity | same | | 0.23 | male age | 0 | 0.02 |
|  |  | |  | male rank | 4.29 | 0.91 |
| Betweenness | same | | 0.34 | female age | 0.97 | 0.52 |
|  |  | |  | female rank | 1.53 | 0.62 |
| Betweenness | same | | 0.54 | male age | 4.7 | 0.9 |
|  |  | |  | **male rank** | **5.33** | **0.95*** |
| Eigenvector Centrality | same | | 0.14 | female age | 3.06 | 0.84 |
|  |  | |  | female rank | 0.94 | 0.48 |
| Eigenvector Centrality | same | | 0.7 | **male age** | **6.06** | **0.99*** |
|  |  | |  | male rank | 2.19 | 0.8 |
|  | |  |  |  |  |  |

**Table S5. Age effects independent of rank and time sexually swollen on female social integration in mixed-sex networks.** Significant effects in bold with*. DE = total model deviance explained. Significance of all smooth terms (age, rank, time swollen, and their interaction) evaluated by the proportion of F statistics drawn from randomized networks that the observed F statistic is greater than.

| **Response** | **Network sex** | **DE** | **Predictors** | **F_obs_ of smooths** | **F_obs_ > F_ran_** |
| --- | --- | --- | --- | --- | --- |
| In-Degree | mixed | 0.53 | Age | 2.9 | 0.87 |
|  |  |  | Rank | 4.5 | 0.47 |
|  |  |  | **Time swollen** | **0.76** | **0.96*** |
|  |  |  | Age * Swollen | 4.07 | 0.94 |
| Out-Degree | mixed | 0.78 | Age | 2.66 | 0.78 |
|  |  |  | Rank | 6.69 | 0.75 |
|  |  |  | **Time swollen** | **2.9** | **0.97*** |
|  |  |  | Age * Swollen | 2.9 | 0.72 |
| In-Strength | mixed | 0.29 | Age | 0.57 | 0.47 |
|  |  |  | **Rank** | **2.71** | **0.97*** |
|  |  |  | Time swollen | 5.58 | 0.88 |
|  |  |  | **Age * Swollen** | **9.66** | **0.99*** |
| Out-Strength | mixed | 0.43 | **Age** | **16.17** | **1*** |
|  |  |  | Rank | 1.3 | 0.48 |
|  |  |  | Time swollen | 0.63 | 0.69 |
|  |  |  | Age * Swollen | 0.25 | 0.28 |
| Betweenness | mixed | 0.25 | Age | 1.99 | 0.8 |
|  |  |  | Rank | 2.23 | 0.4 |
|  |  |  | Time swollen | 0.41 | 0.87 |
|  |  |  | Age * Swollen | 1.41 | 0.71 |
| Local Transitivity | mixed | 0.48 | Age | 0.08 | 0.08 |
|  |  |  | Rank | 1.86 | 0.11 |
|  |  |  | Time swollen | 0.22 | 0.29 |
|  |  |  | Age * Swollen | 1.9 | 0.26 |
| Eigenvector Centrality | mixed | 0.75 | Age | 0.15 | 0.14 |
|  |  |  | Rank | 4.85 | 0.72 |
|  |  |  | Time swollen | 2.35 | 0.84 |
|  |  |  | Age * Swollen | 4.23 | 0.86 |

**Table S6. Repeatability of integration measures by behavior, network type, and sex.** Repeatability statistic calculated by the observed deviance explained by individual ID alone (IDE_obs_) in Generalized Additive Mixed Models (GAMMs). Significance of IDE_obs_ evaluated as the percentage of 1000 deviances explained by ID in GAMMs on node-randomized data (IDE_ran_) that IDE_obs_ is greater than.

| **Behavior** | **Network sex** | **Sex** | **SNA measure** | **IDE_obs_** | **%IDE_obs_ < 1000 IDE_ran_** |
| --- | --- | --- | --- | --- | --- |
| Grooming | mixed | Male | In-Degree | 0.01 | 72 |
|  |  |  | **Out-Degree** | **0.18** | **96*** |
|  |  |  | **In-Strength** | **0.37** | **100*** |
|  |  |  | Out-Strength | 0.12 | 77 |
|  |  |  | Local Transitivity | 0.1 | 77 |
|  |  |  | Betweenness | 0.03 | 40 |
|  |  |  | Eigenvector Centrality | 0.15 | 88 |
| Grooming | mixed | Female | **In-Degree** | **0.21** | **98*** |
|  |  |  | **Out-Degree** | **0.52** | **100*** |
|  |  |  | **In-Strength** | **0.18** | **100*** |
|  |  |  | **Out-Strength** | **0.21** | **100*** |
|  |  |  | Local Transitivity | -0.03 | 1 |
|  |  |  | Betweenness | 0.2 | 62 |
|  |  |  | **Eigenvector Centrality** | **0.63** | **100*** |
| Grooming | same | Male | In-Degree | 0 | 63 |
|  |  |  | **Out-Degree** | **0.22** | **100*** |
|  |  |  | **In-Strength** | **0.26** | **96*** |
|  |  |  | Out-Strength | 0.02 | 51 |
|  |  |  | Local Transitivity | 0 | 9 |
|  |  |  | Betweenness | 0.27 | 82 |
|  |  |  | Eigenvector Centrality | 0.15 | 88 |
| Grooming | same | Female | **In-Degree** | **0.36** | **99*** |
|  |  |  | **Out-Degree** | **0.55** | **100*** |
|  |  |  | In-Strength | 0.19 | 72 |
|  |  |  | **Out-Strength** | **0.13** | **100*** |
|  |  |  | Local Transitivity | 0.06 | 78 |
|  |  |  | Betweenness | 0.25 | **95*** |
|  |  |  | Eigenvector Centrality | 0.03 | 51 |

**Table S7. Summary of general age effects:** Age-related changes in social network integration with shape or arrow describing any significant relationship between age and the given network measure. Effects are not controlling for dominance rank or time swollen. Shape and arrows describe significant relationships between age and a given network measure (see Legend; full model results in Tables S8-9). Dots indicate a non-significant pattern. Shading indicates a difference in significant patterns from rank-independent age models.

| **Integration Measure** | **Males**  **(mixed sex)** | **Males**  **(same sex)** | **Females**  **(mixed sex)** | **Females**  **(same sex)** |
| --- | --- | --- | --- | --- |
| *In-Degree* | **⋅** |  | **∩** | **⋅** |
| *Out-degree* | **⋅** | **⋅** | **∩** | **⋅** |
| *In-Strength* | **⋅** | **∩** | **⋅** | **⋅** |
| *Out-Strength* | **⋅** | **⋅** | **⋅** | ↓ |
| *Local Transitivity* | ↑ | **⋅** | **∩** | **⋅** |
| *Betweenness* | **⋅** | **⋅** | **⋅** | **⋅** |
| *Eigenvector centrality* |  |  | **⋅** | ↓ |

**Legend**: Integration measure ↑ = increases with age, ↓ = decreases with age, = increases and plateaus with age, **∩ =** increases in early to mid-adulthood and decreases in later adulthood

**Table S8.** GAMM models with age alone as a predictor of integration measures in mixed-sex grooming networks. Significant effects in bold with *. DE = total model deviance explained. Significance of the categorical variable sex and smooth term age evaluated by the proportion of ßs and F statistics drawn from randomized networks that the observed ß estimate and F statistic are greater than.

| **Response** | **Network sex** | **DE** | **Predictors** | **F_obs_ of smooths** | **ß_obs_ of sex(M)** | **F_obs_ > F_ran_** | **ß_obs_ > ß_ran_** |
| --- | --- | --- | --- | --- | --- | --- | --- |
| In-Degree | mixed | 0.69 | **sex** |  | **0.74** |  | **1*** |
|  |  |  | **female age** | **7.53** |  | **1*** |  |
|  |  |  | male age | 3.08 |  | 0.91 |  |
| Out-Degree | mixed | 0.8 | **sex** |  | **0.95** |  | **1*** |
|  |  |  | **female age** | **3.89** |  | **0.96*** |  |
|  |  |  | male age | 2.06 |  | 0.84 |  |
| In-Strength | mixed | 0.63 | **sex** |  | **1.45** |  | **1*** |
|  |  |  | female age | 0.57 |  | 0.37 |  |
|  |  |  | male age | 3.18 |  | 0.82 |  |
| Out-Strength | mixed | 0.55 | **sex** |  | **1.53** |  | **1*** |
|  |  |  | female age | 2.33 |  | 0.75 |  |
|  |  |  | male age | 1.88 |  | 0.69 |  |
| Local Transitivity | mixed | 0.3 | sex |  | -0.09 |  | 0.01 |
|  |  |  | **female age** | **6.36** |  | **1*** |  |
|  |  |  | **male age** | **17.11** |  | **1*** |  |
| Betweenness | mixed | 0.53 | **sex** |  | **0.33** |  | **0.95*** |
|  |  |  | female age | 3.95 |  | 0.89 |  |
|  |  |  | male age | 0.64 |  | 0.41 |  |
| Eigenvector Centrality | mixed | 0.83 | **sex** |  | **1.46** |  | **1*** |
|  |  |  | female age | 0.81 |  | 0.55 |  |
|  |  |  | **male age** | **7.46** |  | **0.99*** |  |

**Table S9.** GAMM models with age alone as a predictor of integration measures in same-sex grooming networks. Significant effects in bold with *. DE = total model deviance explained. Significance of the smooth term age evaluated by the proportion of F statistics drawn from randomized networks that the observed F statistic is greater than.

| **Response** | **Network sex** | **DE** | **Predictors** | **F_obs_ of smooths** | **F_obs_ > F_ran_** |
| --- | --- | --- | --- | --- | --- |
| In-Degree | same | 0.41 | female age | 2.37 | 0.88 |
| In-Degree | same | 0.53 | **male age** | **3.88** | **0.97*** |
| Out-Degree | same | 0.63 | female age | 1.98 | 0.8 |
| Out-Degree | same | 0.4 | male age | 0.29 | 0.4 |
| In-Strength | same | 0.27 | female age | 4.51 | 0.87 |
| In-Strength | same | 0.7 | **male age** | **6.2** | **0.96*** |
| Out-Strength | same | 0.3 | **female age** | **50.29** | **1*** |
| Out-Strength | same | 0.35 | male age | 6.59 | 0.94 |
| Local Transitivity | same | 0.1 | female age | 1.58 | 0.76 |
| Local Transitivity | same | 0.01 | male age | 0.66 | 0.5 |
| Betweenness | same | 0.34 | female age | 1.91 | 0.77 |
| Betweenness | same |  |  |  |  |
| Eigenvector Centrality | same | 0.15 | female age | 7.43 | **0.99*** |
| Eigenvector Centrality | same | 0.7 | male age | 6.58 | **0.99*** |

**Supplemental References:**

Almeling, L., Hammerschmidt, K., Sennhenn-Reulen, H., Freund, A. M., & Fischer, J. (2016). Motivational shifts in aging monkeys and the origins of social selectivity. *Current Biology*, *26*(13), 1744–1749. https://doi.org/10.1016/j.cub.2016.04.066

Andersen, K. V. (2013). The problem of embeddedness revisited: Collaboration and market types. *Research Policy*, *42*(1), 139–148. https://doi.org/10.1016/j.respol.2012.05.005

Anderson, C. M. (1986). Female age: Male preference and reproductive success in primates. *International Journal of Primatology*, *7*(3), 305–326. https://doi.org/10.1007/BF02736394

Aplin, L. M., Farine, D. R., Morand-Ferron, J., & Sheldon, B. C. (2012). Social networks predict patch discovery in a wild population of songbirds. *Proceedings of the Royal Society B: Biological Sciences*, *279*(1745), 4199–4205. https://doi.org/10.1098/rspb.2012.1591

Balasubramaniam, K., Beisner, B., Vandeleest, J., Atwill, E., & McCowan, B. (2016). Social buffering and contact transmission: Network connections have beneficial and detrimental effects on Shigella infection risk among captive rhesus macaques. *PeerJ*, *4*, e2630. https://doi.org/10.7717/peerj.2630

Bhattacharya, K., Ghosh, A., Monsivais, D., Dunbar, R. I. M., & Kaski, K. (2016). Sex differences in social focus across the life cycle in humans. *Royal Society Open Science*, *3*(4), 160097. https://doi.org/10.1098/rsos.160097

Bray, J., & Gilby, I. C. (2020). Social relationships among adult male chimpanzees (Pan troglodytes schweinfurthii): Variation in the strength and quality of social bonds. *Behavioral Ecology and Sociobiology*, *74*(9), 112. https://doi.org/10.1007/s00265-020-02892-3

Brent, L. J. N. (2015). Friends of friends: Are indirect connections in social networks important to animal behaviour? *Animal Behaviour*, *103*, 211–222. http://dx.doi.org/10.1016/j.anbehav.2015.01.020

Brent, L. J. N., Franks, D. W., Foster, E. A., Balcomb, K. C., Cant, M. A., & Croft, D. P. (2015). Ecological Knowledge, Leadership, and the Evolution of Menopause in Killer Whales. *Current Biology*, *25*(6), 746–750. https://doi.org/10.1016/j.cub.2015.01.037

Carstensen, L. L. (1992). Social and emotional patterns in adulthood: Support for socioemotional selectivity theory. *Psychology and Aging*, *7*(3), 331–338. https://doi.org/10.1037/0882-7974.7.3.331

Carstensen, L. L., Isaacowitz, D. M., & Charles, S. T. (1999). Taking time seriously: A theory of socioemotional selectivity. *American Psychologist*, *54*(3), 165–181. https://doi.org/10.1037/0003-066X.54.3.165

Coleman, J. S. (1988). Social Capital in the Creation of Human Capital. *American Journal of Sociology*, *94*, S95–S120. https://doi.org/10.1086/228943

Cornwell, B., Laumann, E. O., & Schumm, L. P. (2008). The Social Connectedness of Older Adults: A National Profile. *American Sociological Review*, *73*(2), 185–203. https://doi.org/10.1177/000312240807300201

Cornwell, B., Schumm, L. P., Laumann, E. O., & Graber, J. (2009). Social Networks in the NSHAP Study: Rationale, Measurement, and Preliminary Findings. *The Journals of Gerontology: Series B*, *64B*(suppl_1), i47–i55. https://doi.org/10.1093/geronb/gbp042

Croft, D. P., Madden, J. R., Franks, D. W., & James, R. (2011). Hypothesis testing in animal social networks. *Trends in Ecology & Evolution*, *26*(10), 502–507. http://dx.doi.org/10.1016/j.tree.2011.05.012

David-Barrett, T., Kertesz, J., Rotkirch, A., Ghosh, A., Bhattacharya, K., Monsivais, D., & Kaski, K. (2016). Communication with Family and Friends across the Life Course. *PLOS ONE*, *11*(11), e0165687. https://doi.org/10.1371/journal.pone.0165687

Donald, C. A., & Ware, J. E. (1984). The measurement of social support. *Research in Community & Mental Health*, *4*, 325–370.

Duboscq, J., Romano, V., Sueur, C., & MacIntosh, A. J. J. (2016). Network centrality and seasonality interact to predict lice load in a social primate. *Scientific Reports*, *6*, 22095. https://doi.org/10.1038/srep22095 http://www.nature.com/articles/srep22095#supplementary-information

Emery Thompson, M., Machanda, Z. P., Fox, S. A., Sabbi, K. H., Otali, E., Thompson González, N., Muller, M. N., & Wrangham, R. W. (2020). Evaluating the impact of physical frailty during ageing in wild chimpanzees (Pan troglodytes schweinfurthii). *Philosophical Transactions of the Royal Society B: Biological Sciences*, *375*(1811), 20190607. https://doi.org/10.1098/rstb.2019.0607

English, T., & Carstensen, L. L. (2014). Selective narrowing of social networks across adulthood is associated with improved emotional experience in daily life. *International Journal of Behavioral Development*, *38*(2), 195–202. https://doi.org/10.1177/0165025413515404

Farine, D. R., & Whitehead, H. (2015). Constructing, conducting and interpreting animal social network analysis. *Journal of Animal Ecology*, *84*(5), 1144–1163. https://doi.org/10.1111/1365-2656.12418

Fung, H. H., Carstensen, L. L., & Lang, F. R. (2001). Age-related patterns in social networks among European Americans and African Americans: Implications for socioemotional selectivity across the life span. *The International Journal of Aging & Human Development*, *52*(3), 185–206. https://doi.org/10.2190/1ABL-9BE5-M0X2-LR9V

Glowacki, L., & von Rueden, C. (2015). Leadership solves collective action problems in small-scale societies. *Philosophical Transactions of the Royal Society B: Biological Sciences*, *370*(1683), 20150010. https://doi.org/10.1098/rstb.2015.0010

Goldenberg, S. Z., de Silva, S., Rasmussen, H. B., Douglas-Hamilton, I., & Wittemyer, G. (2014). Controlling for behavioural state reveals social dynamics among male African elephants, Loxodonta africana. *Animal Behaviour*, *95*, 111–119. https://doi.org/10.1016/j.anbehav.2014.07.002

Granovetter, M. (1983). The Strength of Weak Ties: A Network Theory Revisited. *Sociological Theory*, *1*, 201–233. https://doi.org/10.2307/202051

Granovetter, M. (1985). Economic Action and Social Structure: The Problem of Embeddedness. *American Journal of Sociology*, *91*(3), 481–510. https://doi.org/10.1086/228311

Hanneman, R. A., & Riddle, M. (2005). *Introduction to social network methods*. University of California, Riverside.

Holt-Lunstad, J., Smith, T. B., & Layton, J. B. (2010). Social relationships and mortality risk: A meta-analytic review. *PLOS Medicine*, *7*(7), 1–20. https://doi.org/10.1371/journal.pmed.1000316

James, E. H. (2000). Race-Related Differences in Promotions and Support: Underlying Effects of Human and Social Capital. *Organization Science*, *11*(5), 493–508. https://doi.org/10.1287/orsc.11.5.493.15202

Keating, N., Swindle, J., & Foster, D. (2005). The Role of Social Capital in Aging Well. In *Social Capital in Action: Thematic Policy Studies* (pp. 24–50). Policy Research Initiative.

Liao, Z., Sosa, S., Wu, C., & Zhang, P. (2018). The influence of age on wild rhesus macaques’ affiliative social interactions. *American Journal of Primatology*, *80*(2), e22733. https://doi.org/10.1002/ajp.22733

Lusseau, D. (2007). Evidence for social role in a dolphin social network. *Evolutionary Ecology*, *21*(3), 357–366. https://doi.org/10.1007/s10682-006-9105-0

Lusseau, D., & Newman, M. E. J. (2004). Identifying the role that animals play in their social networks. *Proceedings of the Royal Society of London. Series B: Biological Sciences*, *271*(suppl_6), S477–S481. https://doi.org/10.1098/rsbl.2004.0225

Machanda, Z. P., & Rosati, A. G. (2020). Shifting sociality during primate ageing. *Philosophical Transactions of the Royal Society B: Biological Sciences*, *375*(1811), 20190620. https://doi.org/10.1098/rstb.2019.0620

Matthews, G. A., & Tye, K. M. (2019). Neural mechanisms of social homeostasis. *Annals of the New York Academy of Sciences*, *1457*(1), 5–25. https://doi.org/10.1111/nyas.14016

McComb, K., Moss, C., Durant, S. M., Baker, L., & Sayialel, S. (2001). Matriarchs As Repositories of Social Knowledge in African Elephants. *Science*, *292*(5516), 491–494. https://doi.org/10.1126/science.1057895

McComb, K., Shannon, G., Durant, S. M., Sayialel, K., Slotow, R., Poole, J., & Moss, C. (2011). Leadership in elephants: The adaptive value of age. *Proceedings of the Royal Society B: Biological Sciences*.

Mitani, J. C. (2009). Male chimpanzees form enduring and equitable social bonds. *Animal Behaviour*, *77*(3), 633–640. https://doi.org/10.1016/j.anbehav.2008.11.021

Muller, M. N., Thompson, M. E., & Wrangham, R. W. (2006). Male Chimpanzees Prefer Mating with Old Females. *Current Biology*, *16*(22), 2234–2238. https://doi.org/10.1016/j.cub.2006.09.042

Muller, M. N., & Wrangham, R. W. (2004). Dominance, aggression and testosterone in wild chimpanzees: A test of the ‘challenge hypothesis.’ *Animal Behaviour*, *67*(1), 113–123. https://doi.org/10.1016/j.anbehav.2003.03.013

Muller, M. N., & Wrangham, R. W. (2014). Mortality rates among Kanyawara chimpanzees. *Journal of Human Evolution*, *66*, 107–114. https://doi.org/10.1016/j.jhevol.2013.10.004

Nakagawa, S., Johnson, P. C. D., & Schielzeth, H. (2017). The coefficient of determination R2 and intra-class correlation coefficient from generalized linear mixed-effects models revisited and expanded. *Journal of The Royal Society Interface*, *14*(134), 20170213. https://doi.org/10.1098/rsif.2017.0213

Nunn, C. L. (2012). Primate Disease Ecology in Comparative and Theoretical Perspective. *American Journal of Primatology*, *74*(6), 497–509. https://doi.org/10.1002/ajp.21986

Page, A. E., Chaudhary, N., Viguier, S., Dyble, M., Thompson, J., Smith, D., Salali, G. D., Mace, R., & Migliano, A. B. (2017). Hunter-Gatherer Social Networks and Reproductive Success. *Scientific Reports*, *7*(1), 1153. https://doi.org/10.1038/s41598-017-01310-5

Rathke, E.-M., & Fischer, J. (2021). Social aging in male and female Barbary macaques. *American Journal of Primatology*, *n/a*(n/a), e23272. https://doi.org/10.1002/ajp.23272

Schielzeth, H., & Nakagawa, S. (2020). Conditional repeatability and the variance explained by reaction norm variation in random slope models. *BioRxiv*, 2020.03.11.987073. https://doi.org/10.1101/2020.03.11.987073

Silk, J. B. (2007). The adaptive value of sociality in mammalian groups. *Philosophical Transactions of the Royal Society Biological Sciences*, *362*, 539–559. https://doi.org/10.1098/rstb.2006.1994

Sosa, S., Sueur, C., & Puga‐Gonzalez, I. (2020). Network measures in animal social network analysis: Their strengths, limits, interpretations and uses. *Methods in Ecology and Evolution*, *n/a*(n/a). https://doi.org/10.1111/2041-210X.13366

Stowe, J. D., & Cooney, T. M. (2015). Examining Rowe and Kahn’s Concept of Successful Aging: Importance of Taking a Life Course Perspective. *The Gerontologist*, *55*(1), 43–50. https://doi.org/10.1093/geront/gnu055

Thompson, N. A. (2019). Understanding the links between social ties and fitness over the life cycle in primates. *Behaviour*, *156*(9), 1–50. https://doi.org/10.1163/1568539X-00003552

Tokuyama, N., & Furuichi, T. (2017). *Leadership of old females in collective departures in wild bonobos (Pan paniscus) at Wamba*. https://pubag.nal.usda.gov/catalog/5756172

VanderWeele, T. J. (2016). Mediation Analysis: A Practitioner’s Guide. *Annual Review of Public Health*, *37*(1), 17–32. https://doi.org/10.1146/annurev-publhealth-032315-021402

von Rueden, C., Gurven, M., & Kaplan, H. (2008). The multiple dimensions of male social status in an Amazonian society. *Evolution and Human Behavior*, *29*(6), 402–415. https://doi.org/10.1016/j.evolhumbehav.2008.05.001

Weiss, M. N., Franks, D. W., Brent, L. J. N., Ellis, S., Silk, M. J., & Croft, D. P. (2021). Common datastream permutations of animal social network data are not appropriate for hypothesis testing using regression models. *Methods in Ecology and Evolution*, *12*(2), 255–265. https://doi.org/10.1111/2041-210X.13508

Wen Yuan, C., Kropczynski, J., Wirth, R., Rosson, M. B., & Carroll, J. M. (2017). Investigating Older Adults’ Social Networks and Coproduction Activities for Health. *Proceedings of the 11th EAI International Conference on Pervasive Computing Technologies for Healthcare*, 68–77. https://doi.org/10.1145/3154862.3154876

Wey, T. W., & Blumstein, D. T. (2010). Social cohesion in yellow-bellied marmots is established through age and kin structuring. *Animal Behaviour*, *79*(6), 1343–1352.

Wood, S. N. (2017). *Generalized Additive Models: An Introduction with R, Second Edition*. CRC Press.

Wrzus, C., Hänel, M., Wagner, J., & Neyer, F. J. (2013). Social network changes and life events across the life span: A meta-analysis. *Psychological Bulletin*, *139*(1), 53–80. https://doi.org/10.1037/a0028601

Young, C., Majolo, B., Schülke, O., & Ostner, J. (2014). Male social bonds and rank predict supporter selection in cooperative aggression in wild Barbary macaques. *Animal Behaviour*, *95*, 23–32. https://doi.org/10.1016/j.anbehav.2014.06.007
