# Supplementary figures and images for: Age-related change in adult chimpanzee social network integration
Source: Evol Med Public Health. 2021 Dec 1;9(1):448–59. doi: 10.1093/emph/eoab040 (PMC8697844; doi:10.1093/emph/eoab040)

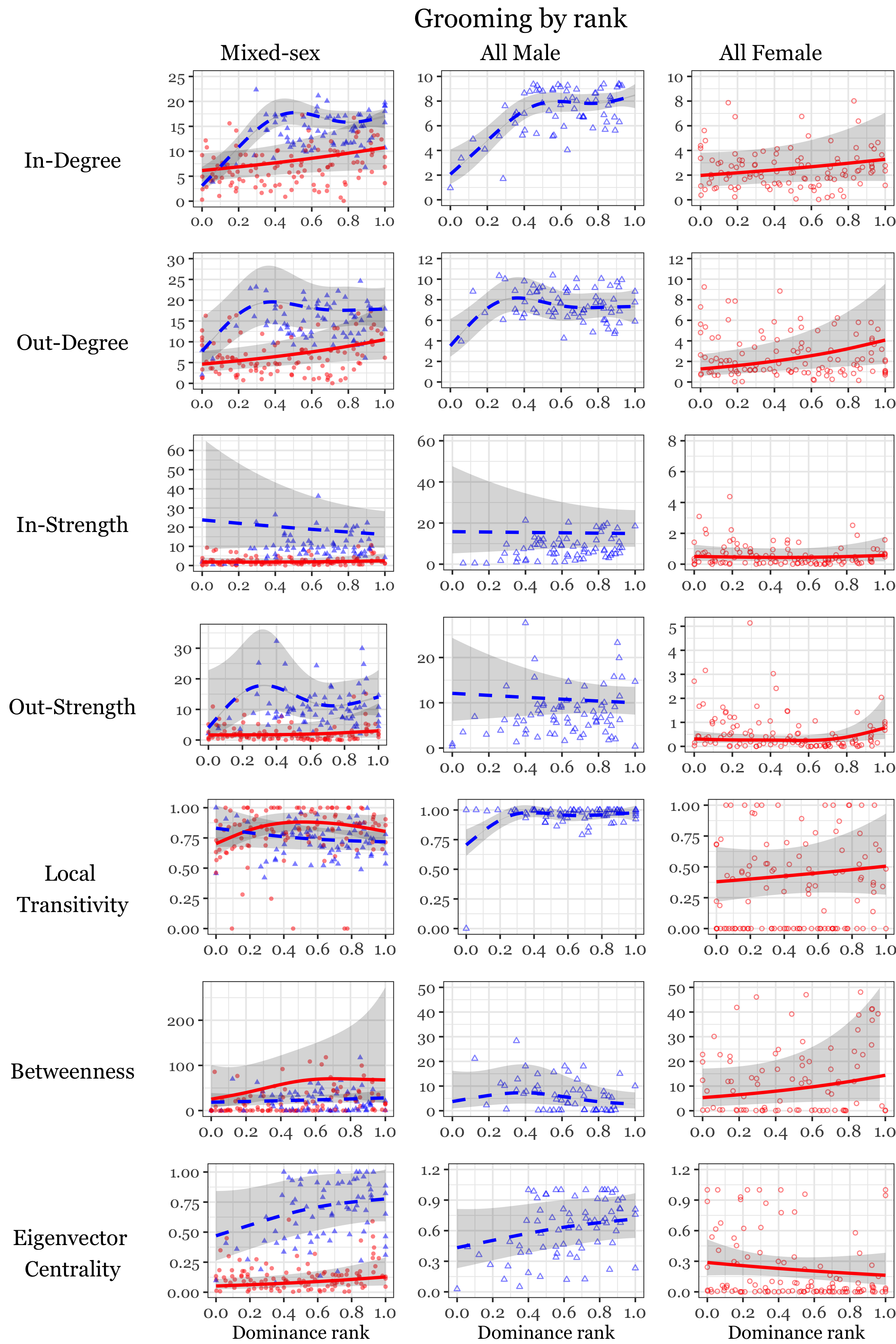

Supplement: eoab040_Supplementary_Data [file eoab040_supplementary_data.zip › Fig S1. Grooming by rank panel.png]

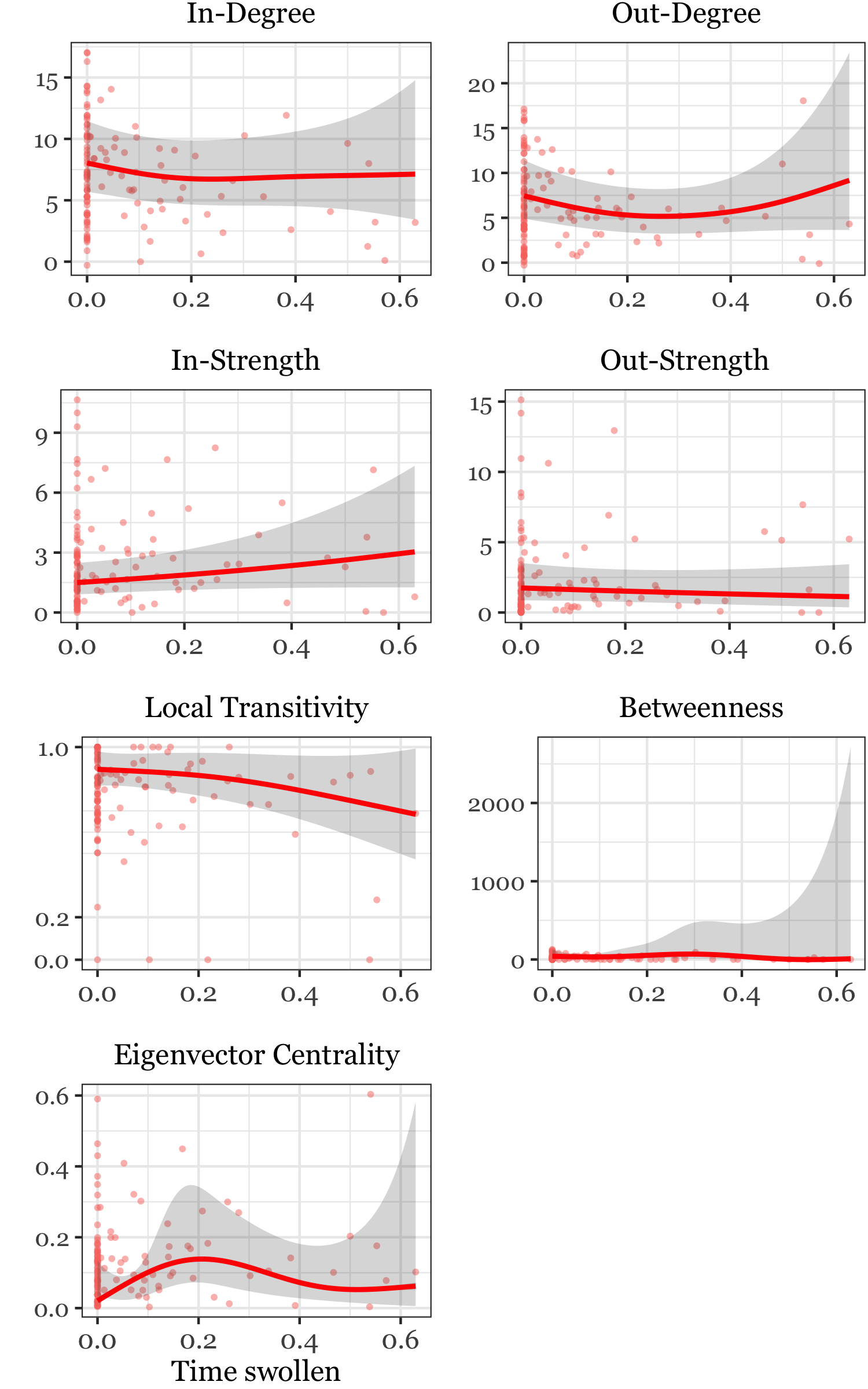

Supplement: eoab040_Supplementary_Data [file eoab040_supplementary_data.zip › Fig S2. female mixed sex sna by time swollen.png]

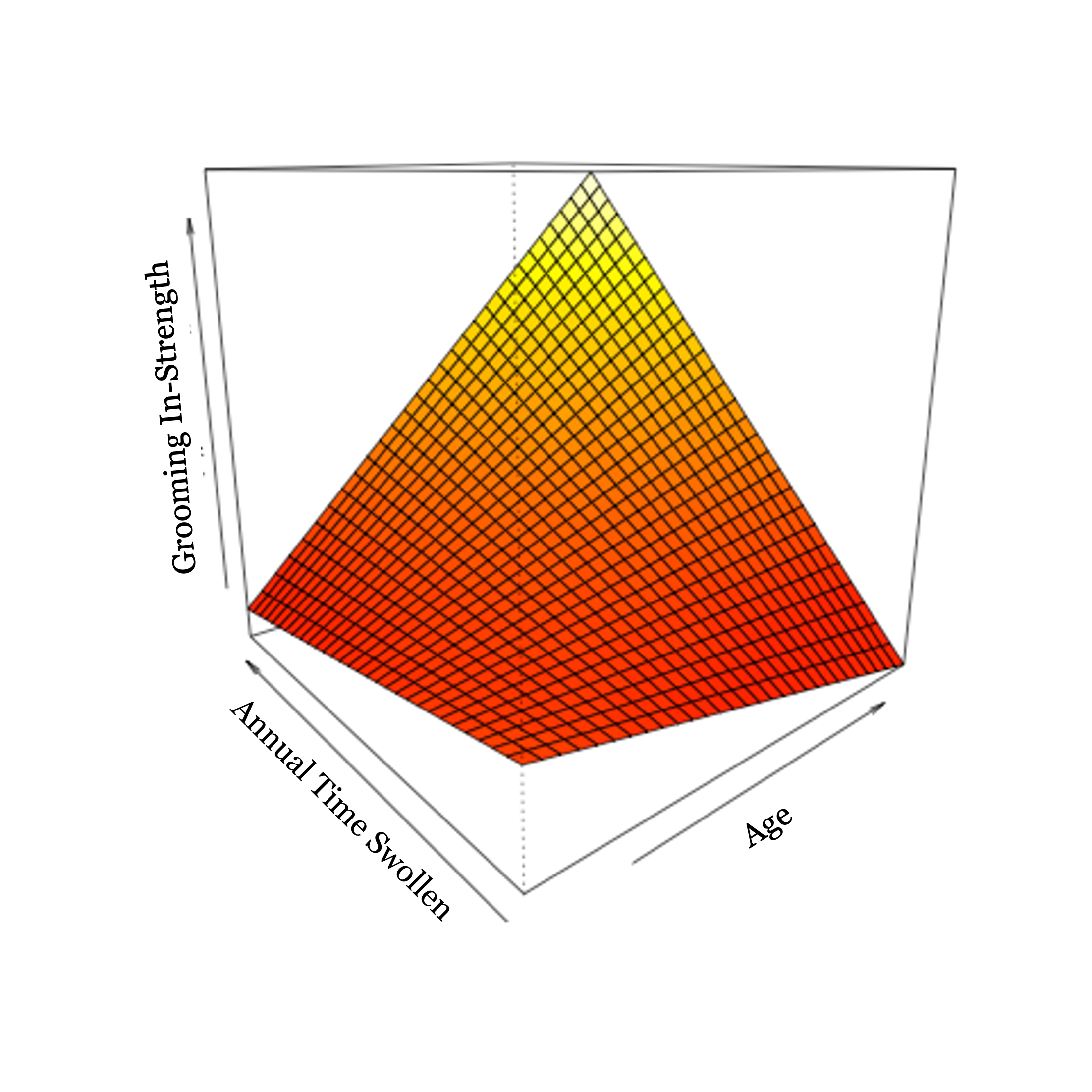

Supplement: eoab040_Supplementary_Data [file eoab040_supplementary_data.zip › Fig S3. strength in prop cyc and age w labels.png]
